# Brain–spine interfaces to reverse paralysis

**DOI:** 10.1093/nsr/nwac009

**Published:** 2022-01-18

**Authors:** Henri Lorach, Guillaume Charvet, Jocelyne Bloch, Grégoire Courtine

**Affiliations:** Center for Neuroprosthetics and Brain Mind Institute, School of Life Sciences, Swiss Federal Institute of Technology (EPFL), Switzerland; Department of Clinical Neuroscience, Lausanne University Hospital (CHUV) and University of Lausanne (UNIL), Switzerland; Defitech Center for Interventional Neurotherapies (NeuroRestore), EPFL/CHUV/UNIL, Switzerland; CEA, LETI, CLINATEC, University Grenoble Alpes, France; Center for Neuroprosthetics and Brain Mind Institute, School of Life Sciences, Swiss Federal Institute of Technology (EPFL), Switzerland; Department of Clinical Neuroscience, Lausanne University Hospital (CHUV) and University of Lausanne (UNIL), Switzerland; Defitech Center for Interventional Neurotherapies (NeuroRestore), EPFL/CHUV/UNIL, Switzerland; Department of Neurosurgery, CHUV, Switzerland; Center for Neuroprosthetics and Brain Mind Institute, School of Life Sciences, Swiss Federal Institute of Technology (EPFL), Switzerland; Department of Clinical Neuroscience, Lausanne University Hospital (CHUV) and University of Lausanne (UNIL), Switzerland; Defitech Center for Interventional Neurotherapies (NeuroRestore), EPFL/CHUV/UNIL, Switzerland; Department of Neurosurgery, CHUV, Switzerland

Various neurotrauma and neurodegenerative disorders alter the communication between the brain and the regions of the spinal cord that control movement. The consequences are permanent motor deficits or even complete paralysis.

The neurons responsible for the production of leg and arm movements are located in the lumbar and cervical regions of the spinal cord, respectively. Epidural electrical stimulation (EES) applied over these regions of the spinal cord can reactivate these neurons [1]. Evidence suggests that EES directly recruits large-diameter afferent fibers where they enter the spinal cord through the dorsal roots. The recruitment of large-diameter afferent fibers leads to the activation of motor neurons embedded in the spinal segment innervated by the root wherein these afferents reside. Since the motor neurons associated with the flexor and extensor muscles of individual joints are located in distinct segments of the spinal cord, targeting an individual dorsal root enables the modulation of specific muscle ensembles.

This understanding translates into stimulation protocols that target the individual dorsal roots with a timing that reproduces the natural spatio-temporal activation patterns of motor neurons underlying the intended movement [2,3]. Spatio-temporal stimulation of the spinal cord has restored standing and walking in people with paralysis due to a spinal-cord injury (SCI) [3]. In these applications, the intended movements were detected using accelerometers and gyroscopes embedded in wearable sensors attached to the lower limbs. This technology enabled transitions from stimulation programs supporting standing and walking, or between the different phases of the gait cycle, but was insufficient to adjust the amplitude of stimulation programs. Consequently, the patients could only exert limited control over the relative activation of muscles, which restricted their ability to accommodate leg movements across activities of daily living. Indeed, the range of residual movements detectable with wearable sensors is limited in patients with incomplete SCI and nearly nonexistent in patients with motor complete SCI.

**Figure 1.**
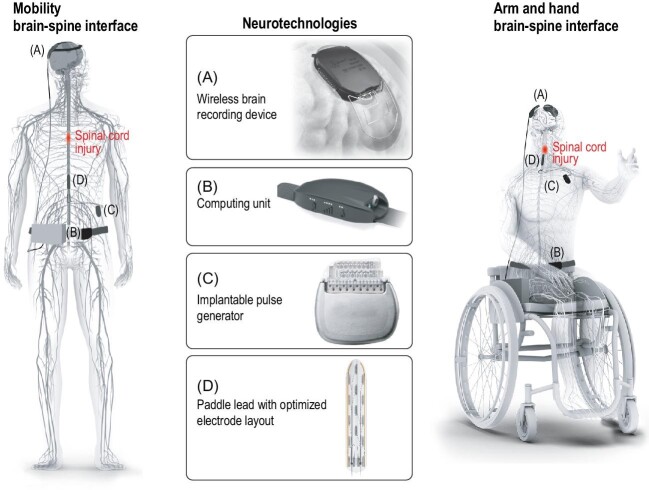
Brain–spine interfaces. The implantable and wearable components of a brain–spine interface are illustrated in the boxes, together with the potential applications to restore mobility and arm/hand functions.

We reasoned that a brain–spine interface (BSI) could remedy these limitations. The underlying idea was to establish a natural link between the brain and spinal cord to enable patients to exert direct control over the protocols of stimulation (Fig. 1).

The implementation of this digital bridge involves several neurotechnological challenges, including the capability to decode motor intentions from neural recordings of the cerebral cortex. Various strategies have been tested to operate neuroprosthetic systems with neural recordings, from non-invasive to highly invasive neurotechnologies [4]. For example, non-invasive recordings of electroencephalography (EEG) signals proved sufficient to link the decoding of movement onset to functional electrical stimulation of upper-limb muscles during neurorehabilitation after stroke and SCI. However, despite innovative developments enabling high-resolution source localization and real-time decoding in a static setting [5], EEG still faces challenges to operate a BSI across mobile activities of daily living since these signals are prone to movement-related artifacts and involve cumbersome hardware. Intracortical microelectrodes inserted into the cerebral cortex resolve this issue. These high-precision recordings enabled patients to operate sophisticated brain–computer interfaces, robotic arms with multiple degrees of freedom, and even functional electrical stimulation of muscles to mobilize paralysed arms [4].

We concluded that validating the concept of BSI in preclinical models would benefit from the highest possible resolution. We therefore selected intracortical microelectrodes to record neural activity from the cerebral cortex. We implanted an intracortical 96-electrode array into the primary motor cortex of nonhuman primates and interfaced this array with an upgraded clinical implantable pulse generator (IPG) enabling real-time control of EES through a wireless bridge. This technology was critical since walking requires the use of untethered systems to enable unconstrained mobility. We thus pioneered a BSI whereby the detection of gait events triggered electrical spinal-cord stimulation protocols that aimed to elicit these events. This BSI restored voluntary control of movements from a paralysed leg in a nonhuman primate model of SCI [2]. We recently expanded this concept to the recovery of upper-limb movements. We interfaced cortical recordings to the modulation of the cervical spinal cord and showed that nonhuman primates with cervical SCI immediately regained volitional control over functional arm movements [6].

These studies have provided critical proofs of concept on the ability of BSIs to restore some degree of control over leg and arm movements after paralysis. Our next objective is to test these concepts clinically. For these applications, we believe that electrocorticographic (ECoG) signals offer the best compromise between invasiveness and spatial resolution. ECoG recordings have been shown to provide sufficient temporal and spatial resolution to decode motor intentions from both leg and arm regions, to remain stable over extensive periods of time, and to withstand movement-related artifacts [7]. Moreover, the recent development of the implantable WIMAGINE system for wireless recordings of ECoG signals provides the necessary technology to deploy a BSI in humans. The challenge will reside in the implementation of decoding algorithms that are robust and can detect motor intentions with latencies compatible with the natural control of movements.

The second key neurotechnology for the design of a clinically viable BSI is an IPG with ultrafast control over multiple stimulation waveforms via wireless links (<50 ms, at least 16 channels). This IPG must be interfaced with a surgical paddle lead that integrates an appropriate density and distribution of electrodes to recruit the individual dorsal roots projecting to the spinal segments embedding the targeted motor neurons. The topology of the dorsal roots differs significantly across the human population, suggesting that a library of paddle leads may be necessary for large-scale deployment of a clinically viable BSI.

The choice of EES technology and epidural electrocorticographic recording will enable long-term use of the BSI system. Indeed, EES has been routinely used to treat chronic pain for >50 years. Stimulation remains stable over decades, only requiring surgical replacements in a minority of cases [8]. The long-term reliability of epidural brain recording is not yet well documented. Yet, these recordings have been reported to remain stable over a period of 32 months [9].

The therapeutic impact of BSI technologies may not be limited to the immediate restoration of movements. Evidence suggests that this type of neuroprosthetic system triggers neuroplasticity of residual nerve connections, which may augment neurological recovery even when the BSI is turned off [10]. For example, lasting improvement of motor functions has been reported in response to brain-actuated neuromuscular stimulation in stroke survivors and people with SCI. Similarly, we showed in preclinical models of SCI that brain-controlled electrical spinal-cord stimulation not only enabled graded control over stimulation parameters to regain walking and stair climbing, but also increased neurological recovery when combined with neurorehabilitation [10].

BSI technologies are amongst the most promising solutions to restore some degree of control over leg and arm movements in people with paralysis. Recent technological breakthroughs in neuroelectronics, signal processing, machine learning, and computational modeling have opened a realistic path to design fully implantable clinical BSIs that could have a real medical, societal and economic impact.
